# The Shortage of Domestic Help

**Published:** 1918-05-11

**Authors:** 


### THE EDITOR'S LETTER-BOX.

### The Shortage of Domestic Help.

*To the Editor of* THE HOSPITAL.

SIR,—Miss Cable, in her letter last week, must, I am sure, have voiced the opinion of many matrons in these trying times. May I offer a suggestion which has been in my mind for some time?

Our civil hospitals are all doing national work of the first importance, and many of them are now taking in soldiers, either acute cases or disabled men. A number of my maids applied to the W.A.A.C., but have been told the Corps will no longer take maids from hospitals. This is very fair and just, but it does not overcome the difficulty of keeping the maids, for they now leave to go into private service with the object of joining the W.A.A.C. from there. My suggestion is that the W.A.A.C. might accept these girls and then put them back to the hospitals they applied from, or to other hospitals, and make them understand that they are doing work of national importance.

Is it not possible that some such arrangement, some sort of amalgamation, might be made with the W.A.A.C.?—I am, yours faithfully,

MATRON OF SCOTTISH HOSPITAL.

